# Reduced Hospitalizations, Emergency Room Visits, and Costs Associated with a Web-Based Health Literacy, Aligned-Incentive Intervention: Mixed Methods Study

**DOI:** 10.2196/14772

**Published:** 2019-10-17

**Authors:** Jeffrey C Greene, Jolie N Haun, Dustin D French, Susan L Chambers, Robert H Roswell

**Affiliations:** 1 MedEncentive Oklahoma City, OK United States; 2 College of Public Health University of South Florida Tampa, FL United States; 3 Department of Ophthalmology Feinberg School of Medicine Northwestern University Chicago, IL United States; 4 Oklahoma City Gynecology & Obstetrics Oklahoma City, OK United States; 5 University of Oklahoma College of Medicine Health Administration and Policy University of Oklahoma College of Public Health Oklahoma City, OK United States

**Keywords:** cost control, health care costs, health literacy, information therapy, aligned incentives, mutual accountability

## Abstract

**Background:**

The association between health literacy and health care costs, particularly for hospitalizations and emergency room services, has been previously observed. Health information interventions aimed at addressing the negative impacts of inadequate health literacy are needed. The MedEncentive Mutual Accountability and Information Therapy (MAIT) Program is a Web-based system designed to improve health and lower costs by aligning patient-doctor incentives.

**Objective:**

In this mixed methods study of a Web-based patient-doctor aligned-incentive, information therapy program conducted in an 1800-member employee health plan, we aimed to (1) determine the program’s quantitative impact on hospitalization and emergency room utilization and costs, and (2) assess survey responses about the program’s perceived value.

**Methods:**

We used a mixed methods, single within-group, pre-post, descriptive study design. We analyzed quantitative data using pre-post mean utilization and cost differences and summarized the data using descriptive statistics. We used open-ended electronic survey items to collect descriptive data and analyzed them using thematic content analysis.

**Results:**

Hospitalizations and emergency room visits per 1000 decreased 32% (26.5/82.4) and 14% (31.3/219.9), respectively, after we implemented the program in 2015-2017, relative to 2013-2014. Correspondingly, the plan’s annual per capita expenditures declined US $675 (95% CI US $470-865), or 10.8% ($675/$6260), after program implementation in 2015-2017 (US $5585 in 2013-2014 dollars), relative to the baseline years of 2013-2014 (US $6260; *P*<.05). Qualitative findings suggested that respondents valued the program, benefiting from its educational and motivational aspects to better self-manage their health.

**Conclusions:**

Analyses suggested that the reported reductions in hospitalizations, emergency room visits, and costs were associated with the program. Qualitative findings indicated that targeted users perceived value in participating in the MAIT Program. Further research with controls is needed to confirm these outcomes and more completely understand the health improvement and cost-containment capabilities of this Web-based health information, patient-doctor, aligned-incentive program.

## Introduction

### Background

Health literacy is defined as the “degree to which individuals have the capacity to obtain, process, and understand basic health information and services needed to make appropriate health decisions” [1] and “the capacity of individuals to obtain, interpret, and understand basic health information and services and the competence to use such information and services in ways which enhance health” [1]. The association between patients’ health literacy levels and hospitalizations, preventable emergency room visits, and overall health care costs is established in the literature [2-7]. Not only is inadequate health literacy harmful and expensive, it is also prevalent. The 2003 National Assessment of Adult Literacy suggests that only 1 in 9 adults in the United States has proficient health literacy [6], contributing to billions of dollars in preventable expenditures per year [7]. Previous population-level studies have confirmed, when controlling for other person-level factors, that lower health literacy is a significant, independent factor associated with increased health care utilization and costs [2,3,5,8-10]. Specifically, inadequate health literacy has been associated with higher rates of hospitalizations and preventable emergency room visits [2,8,9]. Citing overwhelming empirical evidence, the US Department of Health & Human Services designated health literacy improvement as a top priority in 2010 [10]. Although inadequate health literacy is harmful, expensive, and prevalent, there have been few viable solutions to address the effects of inadequate health literacy in the general population and, much less, best practices to narrow the doctor-patient information asymmetry on a group level [5]. Best practices in the field of health literacy have recommended the need for universal precautions [11].

For decades, financial incentives to improve health care and health behaviors have been directed toward physicians and patients separately, with marginal success [12,13]. A recent study, in which a form of patient-doctor, aligned incentives was compared with traditional methods, found that the aligned-incentive approach produced superior outcomes [14]. Based on this finding, leading researchers in the field of behavioral economics concluded that “[aligned] financial incentives for patients and physicians could generate synergies that help patients, physicians, and health insurers achieve greater improvements in population health” [13]. Similar to health literacy, there are few, if any, viable patient-doctor, aligned-incentive solutions.

*Information therapy* is a term for “supplying patients with health information, enabling them to make informed decisions about their health and care, participate in their own well-being, and thus decrease the utilization of healthcare resources” [15]. It is further defined as providing patients with the right information, at the right time, in the right way, so patients can make informed decisions about their health [16]. Compensating physicians to provide an information therapy prescription to their patients as a reimbursable service is a concept suggested in the literature years ago but, heretofore, never attempted in a real-world setting [17]. Incentivizing patients to engage in information therapy and demonstrate assimilation of the information is a new concept, as is the idea of offering patient-doctor aligned incentives to empower and motivate patients with knowledge to self-manage their health. Reward-induced information therapy has the potential to offer a simple and sustainable solution to mitigating the debilitating effects of inadequate health literacy, in a manner that improves health and lowers per capita utilization and expenditures [17,18].

Prior research suggests that the majority of the general population have inadequate or marginal health literacy, requiring remediation when accessing the health care system [6]. Information therapy, as an established, systemwide practice, potentially provides a universal approach to support patients’ health information needs—a key contribution to health literacy. We contend that utilization and cost are established outcome proxies to assess effects associated with information therapy [2,7].

### Objective

The purpose of this study was to evaluate the outcomes of an employee health plan over a 5-year period, before and after the introduction of a Web-based information therapy, patient-doctor, aligned-incentive program. More specifically we aimed to (1) determine the impact (quantitatively) of the program on inpatient and emergency room utilization and costs, which also are proxies for overall health status, before and after implementation; and (2) evaluate participants’ experiences using the program in correlation with the quantitative results.

### The MedEncentive Mutual Accountability and Information Therapy Program

The MedEncentive Mutual Accountability and Information Therapy (MAIT) Program is a Web-based, mobile-enabled, information therapy, patient-doctor aligned-incentive program that promotes patient education and personal accountability, and supports health care cost containment [17,18]. MedEncentive’s customers are health insurance plans sponsored by self-insured employers, governments, health systems, and commercial insurers. The MedEncentive program augments the sponsor’s health plan (*plan*) as an additional benefit to the plan’s members (*beneficiaries*). In the case of self-insured employers, the plan’s summary plan description is modified to recognize the program as a benefit. As part of the service agreement, the plan sponsor directs its plan administrator (third-party administrator) to electronically transmit plan-member demographic enrollment and claims files to MedEncentive’s computer system, and receive reward files for payment to doctors and patients who participate in the program. These electronic data exchanges employ industry-standard transmission protocols and data formats, so that they are secure, automated, and maintenance-free.

#### Program Overview

The MAIT Program uses plan sponsor-supplied member enrollment data to send orientation letters and personalized membership identification cards to all adult plan members. A program *opportunity*, for both doctors and patients, is initiated when doctors access the program’s website, or as a result of MedEncentive’s receipt of a claim associated with a covered service. Covered services include any visit, consultation, or preventive examination rendered in-office to a covered member, by physicians (eg, doctor of medicine, doctor of osteopathy) of any medical specialty, or by licensed physician extenders (ie, nurse practitioners or physician assistants), for any medical condition or wellness examination.

#### The Provider’s Experience

MedEncentive uses the diagnosis from the transmitted office visit claim to notify physicians of program opportunities via fax and email. These notices direct physicians to the MedEncentive website, where they may elect to participate in the program in 2 ways: (1) on a point-of-service (POS)-initiated basis, or (2) on a claims-initiated basis. The POS-initiated version is typically accomplished when practice personnel assign an identifier in their in-office computer system to patients covered by the program. These systems automatically notify doctors to access the program’s website during or shortly after a covered office visit, to initiate an opportunity by entering the patient’s diagnosis (see Multimedia Appendix 1). The MAIT Program can be integrated with in-office systems, which was the case in this implementation.

The claims-initiated version serves as a safety net in case a POS-initiated opportunity is missed. In the claims-initiated version, the MedEncentive system monitors incoming insurance claims to see whether physicians have previously used the POS-initiated version of the program. If not, then the system uses claim information to preload the patient’s diagnosis and send the doctor the fax or email opportunity notice. When doctors choose to participate via the POS- or claims-initiated version, they access the program’s website to complete 2 tasks: (1) consider evidence-based medicine treatment guidelines, and (2) select a patient educational article that the program’s computer system lists in relevancy order to the patient’s diagnosis (see Multimedia Appendix 2). The POS-initiated version functions identically to the claims-initiated version, with the exception of diagnosis input, time limits, and level of compensation. Since the program places a premium on timeliness, doctors earn US $15.00 for completing a POS-initiated session and US $7.50 for completing a claims-initiated session.

When physicians do not participate within 4 days of a claims-initiated notification, patients select their own articles from the list in conjunction with their program opportunity. As a result of this accommodation, both doctors and patients can earn the program’s financial rewards independently of the other party’s participation.

#### The Patient’s Experience

Patient opportunities are initiated as a result of doctor participation, or as a result of office visit claims processing. Patients are notified of their opportunities to participate, by email notices or letters sent to their home, after each office visit (see Multimedia Appendix 3). Patients have 2 weeks to complete their information therapy sessions. For successfully completing a session, patients earn a financial reward, typically a refund of their office visit copay of US $15 or more. To earn their financial reward, patients access the program’s website to (1) read the prescribed or self-selected educational article (see Multimedia Appendix 4), (2) demonstrate their understanding of the health information by passing an open-book test or declaring their comprehension (see Multimedia Appendix 5), (3) declare their adherence or provide a reason for nonadherence (see Multimedia Appendix 6), (4) agree to allow their physician to review their knowledge and adherence assessments (see Multimedia Appendix 7), and (5) rate how consistent their physician’s care is to what they have just learned about recommended treatments (see Multimedia Appendix 8). Participation within required time frames is referred to as an information therapy *success*. When an opportunity expires without completion, this is referred to as a *miss*. Once a quarter, patients are given a second chance to complete the misses that occurred during the previous 90 days.

#### Patient Educational Content

While the program can be adapted to most Web-based educational content, MedEncentive used Healthwise articles in this implementation. Healthwise, Incorporated (Boise, ID, USA) is a conflict-free, nonprofit organization, nationally recognized for providing evidence-based, easy-to-understand health education at the fifth-grade reading level. They supply technology solutions that integrate with complex health information technology systems, with expert guidance on behavior change and shared decision making within the field of health care.

## Methods

### Design

This study used a mixed methods, single within-group, pre-post, descriptive study design to evaluate the MedEncentive MAIT Program. We used open-ended electronic survey items to collect descriptive data. Multimedia Appendix 9 shows the program study flowchart.

### Setting

The study involved the employee health plan of a not-for-profit, acute-care general hospital (health plan sponsor) located in a semirural community in the south-central United States. The hospital is staffed by more than 1400 employees, with more than 100 physicians representing more than 30 specialties.

### Sample

The study sample comprised the plan sponsor’s employees and their covered dependents, to include spouses and children. The employees in the health plan were hospital and clinic personnel, including doctors, nurses, and other medical professionals, as well as administrative and support staff. No person in the covered population was excluded from this study or its analyses. We did not adjust to account for new hires or terminations.

### Data Sources

The analysis of the program implementation relied on multiple sources of data and related background information. The health plan sponsor, its third-party administrator, and its pharmacy benefits manager were the primary sources of plan-member enrollment, medical claims, and pharmacy expenditure data, from 2013 through 2017. We compiled the provider and patient program activity data and survey responses from MedEncentive’s computer system.

#### Quantitative Procedures

Quantifying the impact of the MAIT Program involved a careful, step-by-step process of compiling and evaluating doctor and patient participation rates; the health plan’s 2013-2017 hospitalizations, emergency room visits, and total expenditures; and other demographic and comorbid condition variables, before and after the program was implemented.

#### Doctor and Patient Participation Rates

The MedEncentive computer system automatically calculates doctor and patient participation using a unit of measure called *success rate*. This metric is derived by dividing the number of program successes (successfully completed information therapy sessions) by the total number of opportunities (office visits) incurred by all covered plan members. While patients need to know their doctors have an opportunity to participate—thus making physician inclusion in the program essential—patient success rate is the metric most aligned with reductions in hospitalizations and per capita expenditures at a group level.

#### Hospitalizations, Emergency Room Visits, and Total Health Care Expenditures

Detailed claims data for medical services, excluding pharmacy, were transmitted to MedEncentive on a monthly basis by the plan sponsor and its third-party administrator. Each claim contained more than 200 data elements, such as type of service, diagnosis, rendering provider, service location, gross charges, and net payments. The claims data included physician compensation and patient rewards associated with the program.

We sorted these data by date of service (end date) to organize the medical activity into the year services were rendered, from 2013 through 2017. We observed a typical 90-day run-out period for each year to capture the incurred charges in the year they occurred. We removed dental and optometry claims, since these services are not covered or directly affected by the program. To account for total expenditures, we added the plan members’ direct out-of-pocket payments (copay amount, coinsurance, and deductible) to the amount paid by the plan sponsor. The clinical and economic analyses included all health plan enrollees, before and after implementation, regardless of program participation status.

#### Qualitative Procedures

We collected descriptive data about users’ experiences with the MAIT Program through open- and closed-item electronic surveys. Administered at the conclusion of every information therapy session, for both doctors and patients, the surveys were voluntary and had no effect on the participants’ financial rewards associated with the program. The open-ended items asked physicians and patients about their experience with the program and for their suggestions for improvement. Closed-ended survey items asked patients about (1) how helpful the educational article was for managing their diagnosis or in maintaining their health (2) how closely they were following the health recommendations contained in this article, (3) how much physician access to the program’s survey responses motivated them to improve their health literacy and health behaviors, (4) the importance of their physician’s awareness regarding the patient’s capacity for self-management, and (5) the importance of their physician’s awareness of the patient’s intention to accomplish health objectives.

### Analysis

#### Quantitative Analysis

Since voluntary participation in the program by doctors and patients can be associated with the intended clinical and economic outcomes, we began our quantitative analysis by examining the standard doctor and patient success rate reports generated by the MedEncentive computer system. We were particularly interested in determining whether we had achieved the 55% patient success rate threshold, since it is predictive of the clinical and economic group-level effectiveness of the program.

We analyzed clinical and economic outcomes by comparing annual hospitalizations per 1000 enrollees, emergency room visits per 1000 enrollees, total expenditures per capita, and other variables for 2015-2017 (the implementation period) versus the baseline years (2013-2014), prior to implementing the program. We conducted pre-post analysis of mean cost differences, with confidence intervals, for emergency room, hospitalization, and total care costs [19-22].

Since the program was designed to motivate adherence to recommended treatments and mitigate the effects of inadequate health literacy, which are associated in the literature with hospitalizations, emergency room visits, and total expenditures, these measures were the most effective means to measure program effectiveness [23-26]. To compare annual preimplementation versus postimplementation per capita expenditures, we multiplied the post period (2015-2017) annual episodes (units) of care (hospitalizations, emergency room visits, outpatient services, and pharmacy scripts) by the annual unit costs incurred during the baseline period (2013-2014). The normalization adjustments were made in consultation with the health plan sponsor (hospital) to adjust for known variables, such as pricing, coding, and charge capture. There were no significant benefit design changes over the 5-year period. We considered other health improvement and cost-containment initiatives and, upon analysis, ruled them out as significant contributors to the outcomes analyzed (hospitalizations and emergency room utilization, and total costs).

#### Qualitative Analysis

Structured, open-ended survey-item data were deidentified and cleaned, and prepared for analysis. We managed qualitative survey data responses using thematic content analysis, based on the topic addressed in the structured items and the response (eg, adherence, program satisfaction). We analyzed data in 2 stages to identify domains and taxonomies related to participant experiences [27]. The first round of coding included summarizing and reducing data into preliminary metadomains. Methods included deductive structural coding and inductive descriptive coding, based on themes that emerged from the surveys. In a second round of coding, we reduced coded data into meaningful domains. As we developed coding schemas to create domains, data samples were extracted and coded by at least two team members and evaluated for interrater reliability and validity.

Ultimately, we sorted open-ended survey data into program, provider (doctor), health status, and other categories. Comments pertaining to the program were coded as testimonials, suggestions and service requests, and complaints. Comments pertaining to providers were coded as testimonials and complaints. Comments related to the patient’s health status were coded as general medical condition, improving medical condition, and worsening medical condition. Other comments included insurance complaints.

## Results

### Quantitative Sample-Based Findings

Table 1 presents the health plan’s total enrollees per annum, the mean annual enrollment, and the number of enrollees who received health care, as well as demographic variables, for the years 2013 through 2017. This study’s sample of patients comprised the plan sponsor’s employees and their covered dependents (ie, spouses and children), with a mean of 1803 per year over the 5-year study period (Table 1). It is notable that the plan grew from a mean of 1660 enrollees in 2013 to 1960 enrollees in 2017. The number of plan members receiving care grew from 1560 in 2013 to 1863 in 2017, and the total number of plan members enrolled at any point during a calendar year grew from 1752 in 2013 to 2554 in 2017. This growth was due, in large part, to the expansion of the hospital’s services and acquisition of local medical clinics.

#### Patient Success (Participation) Rates

Patient participation exceeded the targeted 55% success rate threshold in the first year, reaching 68.67% (4245/6182) at the end of 2015. Patient success rate continued to climb to 74.23% (5108/6881) in the first quarter of 2018 (see Multimedia Appendix 10). This level of patient participation predicted, with a high probability, that the clinical and economic outcome objectives would be achieved.

As Table 2 shows, young adults (18-29 years) had the lowest patient success rate (1885/2969, 63.49%) over the 3-year intervention period, while senior adults (≥65 years) had the highest success rate (697/894, 78.0%). Also, the 60- to 65-year age group had the greatest improvement in success rate, climbing 18.4% from 2015 to 2017. This suggests that those with the greatest need participated in the program most frequently and dispels the notion that older adults may be technology challenged or averse to Web-based health literacy mechanisms. Also notable, males and females participated at essentially the same rate (4544/6463, 70.31% vs 8700/12,212, 71.24%, respectively), though females made far more office visits, and the number of office visits per capita remained consistent over the 3-year period (3.3-3.4 visits per annum).

#### Provider Success (Participation) Rates

The overall annual provider success rate started at 30.62% (1890/6173) at the end of 2015 and climbed to 45.41% (2619/5768) by the end of 2016 (see Multimedia Appendix 10). The providers employed by the hospital (health plan sponsor) achieved an even higher level of provider engagement, reaching 55.34% (1654/2989) by the end of 2016. This was due to two developments during the first 18 months of implementation. First, the hospital (plan sponsor) changed its policy of retaining the program’s compensation earned by its employed providers, agreeing to pass these payments on to their participating physicians. Second, the hospital integrated the program with the hospital’s electronic health record system, NextGen, a leading electronic health record supplier. This project was completed in 2016. As a result, doctors were able to access the program through the hospital’s electronic health record system, while having patient demographic and diagnosis information directly transmitted to the MedEncentive computer system. Providers were also automatically notified of covered patients before, during, and immediately after an office visit, offering physicians greater opportunity to use the more timely and higher-paying real-time version of the program.

### Clinical and Economic Outcomes

Program effectiveness can, in part, be measured by an improvement in clinical outcomes, including overall hospitalization and emergency room visit rates. We compiled the total hospitalizations and emergency room visits from the claims data for the 2 years prior to program implementation (2013-2014), and the 3 years after deployment (2015-2017), as Table 3 shows. All members enrolled in the health plan were included in the totals. As Multimedia Appendix 11 illustrates, 2013 and 2014 hospitalizations per 1000, before the introduction of the program, were 87.3 and 82.4, respectively. In 2015, 2016, and 2017, after program implementation, the hospitalization rates were 57.2, 53.9, and 56.6, respectively (*P*<.05). On average, this represents a 32% (26.5/82.4) decrease in admissions per 1000, relative to the baseline year of 2014. Emergency room visits per 1000 in 2013 and 2014 were 251.8 and 219.9, respectively, whereas in 2015, 2016, and 2017, emergency room visits per 1000 plan members decreased to 191.3, 187.5, and 187.3, respectively (*P*<.05). In summary, hospitalizations and emergency room visit rates per 1000 decreased 32% (26.5/82.4) and 14% (31.3/219.9), respectively, in 2015-2017 after implementation of the program, relative to 2013-2014, prior to program implementation, inclusive of all enrollees (participants and nonparticipants).

**Table 1:** Demographic characteristics of patients from 2013-2014 (before program implementation) and 2015-2017 (after program implementation).

Variables		Preimplementation		Postimplementation		
		2013	2014	2015	2016	2017

**Enrollment, n**						
	Total receiving care^a^	1560	1619	1609	1729	1863
	Total enrollees during year^b^	1752	1819	2205	2265	2554
	Mean annual enrollment^c^	1660	1760	1783	1856	1960
**Link to employee-based enrollment, n (%)**						
	Self^b^	744 (42.5)^c^	780 (42.9)	943 (42.9)	964 (42.6)	1065 (41.70)
	Dependent^b^	1008 (57.53)^c^	1039 (57.12)	1262 (57.23)	1301 (57.44)	1489 (58.30)
**Sex, n (%)**						
	Male	748 (42.7)^b,c^	776 (42.7)^a^	944 (42.9)^a^	965 (42.6)^a^	1103 (43.19)^a^
	Female	1004 (57.30)^b,c^	1043 (57.30)^a^	1261 (57.20)^a^	1300 (57.40)^a^	1451 (56.81)^a^
**Age group (years)** ^b^ **, n (%)**						
	0-17	454 (25.9)^c^	468 (25.7)	542 (24.6)	560 (24.7)	630 (24.7)
	18-29	304 (17.4)^c^	350 (19.2)	474 (21.5)	486 (21.5)	582 (22.8)
	30-39	295 (16.9)^c^	298 (16.4)	377 (17.1)	373 (16.5)	392 (15.3)
	40-49	257 (14.7)^c^	277 (15.1)	308 (14.0)	339 (15.0)	432(16.9)
	50-59	274 (15.7)^c^	276 (15.2)	313 (14.2)	313 (13.8)	302 (11.8)
	60-64	104 (6.0)^c^	102 (5.6)	121 (5.5)	123 (5.4)	142 (5.6)
	65	62 (4)^c^	50 (3)	70 (3)	71 (3)	74 (3)
Age (years), mean^b^		33.0^c^	32.8	32.6	32.7	32.5
Office visits per person per year, mean^b^		2.9	3.0	2.9	3.0	3.2

^a^Total members treated during the year.

^b^Total plan-member enrollees during the year.

^c^2013 total enrollees extrapolated from members treated.

**Table 2:** Total patient success rate, total office visits, and overall success percentage (2015-2017), and 3-year trend by demographic characteristics.

Demographic variable		Patient success (n)	Total office visits (n)	Overall success (%)	Change 2015-2017 (%)

**Age group (years)**					
	0-17	2876	3980	72.26	2.23
	18-29	1885	2969	63.49	9.81
	30-39	2235	2987	74.82	1.60
	40-49	2125	2987	71.15	7.82
	50-59	2218	3054	72.63	1.12
	60-64	1208	1804	66.96	18.39
	≥65	697	894	78.0	–6.0
	Age total	13,244	18,675	70.92	4.88
**Relationship**					
	Employee	6771	9361	72.33	4.05
	Dependent	6473	9314	69.50	5.66
	Relationship total	13,244	18,675	70.92	4.88
**Sex**					
	Male	4544	6463	70.31	1.93
	Female	8700	12,212	71.24	6.46
	Sex total	13,244	18,675	70.92	4.88

**Table 3:** Hospitalizations and emergency room visits from 2013-2014 (before program implementation) and 2015-2017 (after program implementation).

Variables	Preimplementation		Postimplementation			
	2013	2014	2015	2016	2017	Postperiod mean

Mean annual enrollment, n	1660	1760	1783	1856	1960	1866
Hospital admissions, n	145	145	102	100	111	104
Admissions per 1000, n	87.3	82.4	57.2	53.9	56.6	55.9
Admissions per 1000 change from 2014, %	N/A^a^	N/A	–30.6	–34.6	–31.2	–32.1
Emergency room visits, n	418	387	341	348	367	352
Emergency room visits per 1000, n	251.8	219.9	191.3	187.5	187.3	188.6
Emergency room visits per 1000 change from 2014, %	N/A	N/A	–13.0	–14.7	–14.8	–14.2

^a^N/A: not applicable.

As Multimedia Appendix 12 illustrates, the plan’s annual per capita expenditures, inclusive of all program costs, declined US $675 (95% CI US $470-865), or 10.8% ($675/$6260), after program implementation in 2015-2017 (US $5585 in 2013-2014 dollars), relative to the baseline years of 2013-2014 (US $6260; *P*<.05), inclusive of all enrollees (participants and nonparticipants) (see Table 4).

**Table 4:** Health care costs from 2013-2014 (before program implementation) and 2015-2017 (after program implementation).

Variables		Preimplementation		Postimplementation			
		2013	2014	2015	2016	2017	Postperiod mean

Total expenditures (all medical and pharmacy)^a^, US $		9,940,434	11,468,059	9,462,011	10,580,146	10,726,060	10,256,072
Total program costs^b^, US $		N/A^c^	N/A	156,403	165,577	179,207	167,062
Annual mean enrollment, n		1660	1760	1783	1856	1960	1866
**Costs per member per year, US $**							
	Total expenditures without program costs^a^	5988	6516	5307	5701	5472	5495
	Total program costs^b^	N/A	N/A	88	89	91	90
	Total expenditures with program costs^a^	5988	6516	5395	5790	5564	5585
	Mean baseline (2013-2014) expenditures	N/A	N/A	6260	6260	6260	6260
	Gross savings^a,d^	N/A	N/A	953	559	787	764
	Net savings^a,e^	N/A	N/A	865	470	696	675
Net savings, %		N/A	N/A	13.8	7.5	11.1	10.8

^a^2015-2017 amounts adjusted to 2013-2014 basis.

^b^N/A: not applicable.

^c^Total program costs for 2015-2017 include all patient rewards, physician compensation, and program administration fees.

^d^Gross savings for 2015-2017=2015-2017 average expenditures less program costs – 2013-2014 average expenditures.

^e^Net savings for 2015-2017=2015-2017 average expenditures with program costs – 2013-2014 average expenditures.

### Quantitative Survey-Based Findings

During 2015-2017, the participating health plan members rated the helpfulness of the program’s educational content at 4.40 out of 5 (with 5 being most helpful), representing 15,260 responses. These ratings indicated there was a strong consensus among patients that the program’s educational content was helpful in managing their disease or condition, or in maintaining good health.

When patients were asked to report (to their doctors) their level of adherence with the health recommendations contained in the program’s educational content, on a scale of 1 to 5, with 1 meaning “not following recommendations” and 5 meaning “following recommendations closely,” the mean response was 4.70 (n=15,186) over the 2015-2017 time period. These self-assessments indicated a strong consensus among patients that they were, or intended to be, compliant with recommended treatments. Table 5 presents survey-item results.

**Table 5:** Patient responses to 5-point Likert-type scale survey items.

Survey item	Response option					Mean
	1	2	3	4	5	

How helpful has this article’s information been to you in managing your disease or condition, or in maintaining your good health? n (%)	187 (1.2)	256 (1.7)	1511 (9.95)	4575 (30.13)	8731 (57.49)	4.40
Please share with your doctor how closely you are following the health recommendations contained in this article as you understand them. n (%)	42 (0)	29 (0)	155 (1.0)	3972 (26.03)	10,988 (72.01)	4.70

The survey item reflecting physician influence on the patient’s motivation to gain health knowledge and improve health behaviors had a mean score of 8.80 out of 10, representing 13,401 responses, indicating a strong consensus that physicians positively influenced patients to improve their health literacy and health behaviors. When asked whether it was important for his or her physician to know of the patient’s competency to self-manage, patient responses had a mean score of 9.24 out of 10, representing 13,401 responses. This indicated that the majority of patients thought it important that their doctor was aware that they understood how to manage their health. Finally, when asked whether it was important that their physicians knew that they were accomplishing health objectives, patient responses had a mean score of 9.26 out of 10, representing 13,401 responses. This indicated that the majority of patients thought it important that their doctor was aware that they were trying to accomplish health objectives. Table 6 presents survey response distributions for these items.

**Table 6:** Patient participant responses to 10-point Likert-type scale survey items.

Survey item	Response level (10=Most)										Mean
	10	9	8	7	6	5	4	3	2	1	

…how much does the knowledge that your physician has access to your questionnaire responses motivate you to improve your health literacy and health behaviors? n (%)	7419 (55.4)	2163 (16.1)	1580 (11.8)	653 (4.9)	552 (4.1)	567 (4.5)	71 (0.5)	99 (0.7)	61 (0.5)	197 (1.8)	8.8
…how important is it to you that your doctor is aware that you understand how to self-manage your health? n (%)	8488 (63.3)	2355 (17.6)	1376 (10.3)	495 (3.7)	290 (2.2)	250 (1.9)	34 (0.3)	30 (0.2)	21 (0.2)	62 (0.5)	9.2
…how important is it to you that your doctor is aware that you are trying to accomplish or are accomplishing health objectives? n (%)	8599 (64.2)	2334 (17.4)	1337 (10.0)	455 (3.4)	280 (2.1)	245 (1.8)	26 (0.2)	39 (0.3)	24 (0.2)	62 (0.5)	9.3

### Qualitative Survey-Based Findings

During 2016-2017, patients posted 555 comments, with 323 (58.2%) pertaining to the program, 183 (33.0%) pertaining to their provider, 31 (5.6%) pertaining to health-related topics, and 18 (3.2%) pertaining to other general topics. Of the program-related comments, the majority (210/323, 65.0%) were testimonials, 33.4% (n=108) were suggestions or requests to improve the program, and 1.6% (n=5) were complaints. Table 7 lists exemplar patient and provider comments about the program.

**Table 7:** Exemplar patient and provider information therapy program-related comments.

Respondent type	Exemplar comments
Patient	Program is great – [puts] a good emphasize on personal accountability.
	Thank you for this MedEncentive program for us. It helps me a lot to know the cause, symptoms, prevention, medicine etc. of my illness. I appreciate it, that my employer has this kind of medical program for their employees.
	I like well enough that I come back later and read related articles.
	I think the more information the patient has the better. You’re taking the right approach in educating the patient. Thank you!
	Excellent plan to get people to take ownership of their health status.
	This is [a] great incentive for patients to not only be aware of their own health and medication issues, but also an opportunity for them to read and learn more about these issues through a formal method of information retrieval. Many adults (old and young) rely upon internet to diagnose and learn about health issues and medications. This format is associated with health professionals and would seem to contain more reliable information and there is financial incentive to completion.
	I love the program. It is educational and beneficial. The financial incentive helps our family greatly as we use it for copays and supplies. We are very thankful for the program.
	I read articles on weight management, and boosting metabolism through exercise and dietary control. I found the sections on boosting metabolism by exercising vigorously at least 2.5 hours weekly in suggested increments, and the reasons for that to be interesting. I feel this is valuable, because I intend to bring my weight under better control.
	Good reminder. It’s hard to remember everything discussed at the appt. this gives me a refresher to read on my own time.
Provider	Keeps me on my toes to talk to patients about diet and exercise.
	The educational handouts are easy to read and are very helpful for my patients. It helps me educate my patients.
	...good selection of articles, easy to prescribe, keep up the good work.
	This is one of the best and most expedient ways of reinforcing discussions we have with our patients in the office setting.

## Discussion

### Principal Findings

The MedEncentive MAIT Program is, to our knowledge, one of the first population-level solutions to use a Web-based approach, combining doctor and patient aligned incentives and information therapy, aimed at improving health and lower costs. Our analysis suggests that the MedEncentive MAIT Program was associated with meaningful reductions in health care utilization that were sustained into the third year of program implementation, through reductions in per capita expenditures and hospitalizations and emergency room use. These findings are in line with previous research on aligned-incentive program expenditure outcomes [18].

Our quantitative analysis found that, from 2015 through 2017, after the program was introduced, hospitalizations and emergency room visits per 1000 plan members, and per capita expenditures, declined relative to the 2013-2014 preimplementation period, by 32.1%, 14.2%, and 10.8%, respectively. Correspondingly, the qualitative survey items suggested that the majority of respondents found the program’s educational content to be very helpful in managing their disease or condition, and maintaining good health. Since patient adherence is such an important predictor of health status, service utilization, and costs [28], it is compelling that the surveys indicated that most patients intended to be compliant with recommended treatments. Furthermore, the surveys found that physicians positively influenced patients to improve their health literacy and health behaviors.

These findings indicated that it was important to patients for their doctor to be aware that they (1) understand how to self-manage their health, and (2) are trying to accomplish health objectives. These reported attitudes help explain the quantitative outcomes and set the stage for aligned-incentive programs to leverage information therapy as a means to mitigate the impact of inadequate health literacy. It is notable that other health improvement initiatives were launched by the plan sponsor, principally in the 2016-2017 time frame. However, the most significant improvement in hospitalizations and emergency room rates was temporally associated with the introduction of the program in 2015.

### Programmatic Implications

Program adoption and retention are predicated on its ease of implementation and maintenance. Over the test period, the plan sponsor experienced continuous program access 99.8% of the time, with no reports of degradation in the program’s website performance due to scaling or spikes in activity. The plan sponsor integrated the program with its clinic electronic health record system to streamline the provider experience. This integration functioned without difficulty throughout program implementation.

The effectiveness of any wellness, prevention, or managed-care program relies on high levels of patient and medical provider engagement in aspects of the program designed to improve health and health care. As the program participation statistics indicate, this goal was achieved and sustained, aided by the collaborative efforts of the plan sponsor and MedEncentive.

### Study Limitations

Study limitations should be considered when interpreting our findings. First, though these findings are compelling, they can only be generalized to not-for-profit, acute-care, general-hospital employee health plans, located in the south-central United States. Second, the primary limitation is the internal nature of the evaluation. Future research should focus on external review and validation. Third, while this study offers associated confirmation of the program’s effectiveness, it is not a randomized control trial and, therefore, falls short of the gold standard for determining causation; hence, further research is needed. Fourth, the conservative analysis may be considered a limitation, compared with more complex analyses (eg, quasi-experimental designs); however, this was prohibited so as to remain compliant with institutional review board standards. Fifth, this evaluation did not control for individual health literacy, but we contend that, statistically, the majority of the general population have inadequate or marginal health literacy and require health information support, such as information therapy; therefore, this program supports health literacy needs at the universal level and measures relevant and meaningful associated outcomes. Future research should control for health literacy. Sixth, we made adjustments to normalize postimplementation expenditures to baseline levels in consultation with the plan sponsor. These adjustments reflect known variances in medical services coding, pricing, charge capture, and benefit design. The precision of these adjustments is difficult to judge, which is further justification to test the program’s capabilities by means of randomized control trials. Finally, although this was an opt-in study design, loss to follow-up can be an issue in cohort studies. Our study, however, included patients who used care throughout the study time frame.

### Conclusions

Use of the Web-based MedEncentive MAIT Program was associated with a reduction in hospitalizations (26.5/82.4, 32%) and emergency room visits (31.3/219.9, 14%) per 1000 members. The plan’s annual per capita expenditures declined US $675 (95% CI US $470-865), or 10.8% ($675/$6260), after program implementation in 2015-2017 (US $5585 in 2013-2014 dollars), relative to the baseline year of 2014 (US $6260; *P*<.05). Our qualitative analysis of participant survey responses corroborated these findings. Therefore, it is reasonable to conclude that the effectiveness of the program was evident in this study. Findings warrant investment in larger, longer-running randomized control trials to further examine and validate these results.

## Appendix

Multimedia Appendix 1 Patient’s diagnosis entered in the program’s website by the doctor.

Multimedia Appendix 2 Doctors select relevant education for their patients.

Multimedia Appendix 3 Patients are notified of their “opportunity” to earn a financial reward for participating in the program.

Multimedia Appendix 4 Patients read educational article specific to their health.

Multimedia Appendix 5 Patients answer questions to confirm their understanding of how to self-manage their health.

Multimedia Appendix 6 Patients declare their adherence with recommended treatments, or provide a reason for nonadherence.

Multimedia Appendix 7 Patients allow their physicians access to their knowledge assessment and adherence declaration.

Multimedia Appendix 8 Patients rate how consistent their physician’s care is to recommended treatments.

Multimedia Appendix 9 MAIT Program study flow diagram.

Multimedia Appendix 10 Patient and provider success rates.

Multimedia Appendix 11 Hospitalization and emergency room rates.

Multimedia Appendix 12 Economic outcomes of the health plan, pre- and post-MAIT Program implementation.

## Abbreviations

MAIT: Mutual Accountability and Information Therapy
POS: point-of-service
