# Supplementary material for: Reduced Hospitalizations, Emergency Room Visits, and Costs Associated with a Web-Based Health Literacy, Aligned-Incentive Intervention: Mixed Methods Study
Source: J Med Internet Res. 2019 Oct 17;21(10):e14772. doi: 10.2196/14772 (PMC6823604; doi:10.2196/14772)
Supplement: Multimedia Appendix 1 [file jmir_v21i10e14772_app1.pdf]

MedEncentive Provider Website

Ix® Activity | Patient Listing ▾ | Profile | Staff Delegation | Logout | Help

Home / Start A New Ix / Patient Identification / Date of Patient Encounter / Patient's Ix Program Activity / **Add Diagnosis**

Enter the Patient's Primary Diagnosis for this Encounter

Patient Name: **Test Patient**
Date of Service: 9/6/2019

To search by the diagnosis code or the diagnosis description, and then enter at least the first three digits of the code or the first three characters of the description.

Diagnosis Code (ICD10) (requires first 3 digits)

_ _ _ . _ _ _

Search for diagnosis code

"or"

Diagnosis Description (Minimum of 3 characters)

Search for keyword

Innovator of the Year Winner 2005, 2006, 2008, 2009, 2013, 2014, and 2016 | U.S. Patents 7,925,519 and 9,171,285 | Canadian Patent 2,729,553

© 2019 MedEncentive, LLC. All Rights Reserved. Privacy Statement

© 2019 MedEncentive, LLC. All Rights Reserved
