# Supplementary material for: Reduced Hospitalizations, Emergency Room Visits, and Costs Associated with a Web-Based Health Literacy, Aligned-Incentive Intervention: Mixed Methods Study
Source: J Med Internet Res. 2019 Oct 17;21(10):e14772. doi: 10.2196/14772 (PMC6823604; doi:10.2196/14772)
Supplement: Multimedia Appendix 2 [file jmir_v21i10e14772_app2.pdf]

MedEncentive Provider Website

Ix® Activity | Patient Listing | Profile | Staff Delegation | Logout | Help

Home / Start A New Ix / Patient / Date of Encounter / Diagnosis(es) / **Prescribe Articles** / Summary

This is the information therapy prescription page

Patient Name: Test Patient
Date Of Service: 9/6/2019
Diagnosis: i10 | Essential Hypertension

Listed in the table below are patient educational articles in order of relevancy to the diagnosis above. Check the box next to one or more articles you wish to prescribe to this patient, and then click "Prescribe the article(s) I selected, below." (**Note:** Be careful not to overwhelm your patient with too many articles all at once.)

Prescribe the article(s) I selected, below

Relevant Articles | My Favorite Articles | Patient's Previous Articles | Wellness Articles | Search for Articles

(**Note:** Additional educational content can be found under the tabs labeled My Favorite Articles, Patient Previous Articles, Wellness Articles, and Article Search.)

Check	Preview	Article Titles Listed in Relevancy Order	Article ID
☐	Preview	High Blood Pressure	hw62787
☐	Preview	Malignant High Blood Pressure	zp3151abc
☐	Preview	High Blood Pressure: Nutrition Tips	[illegible]
☐	Preview	Diabetes: Lower Your Risk for Heart Attack and Stroke	[illegible]
☐	Preview	Blood Pressure Numbers: When to Get Help	[illegible]
☐	Preview	High Blood Pressure: Over-the-Counter Medicines to Avoid	abq1040
☐	Preview	Heart-Healthy Eating	av2065
[illegible]	[illegible]	[illegible]	[illegible]

Doctor selects an educational article for their patient from a list displayed in relevancy order to the patient's diagnosis

© 2019 MedEncentive, LLC. All Rights Reserved
