# Supplementary material for: Reduced Hospitalizations, Emergency Room Visits, and Costs Associated with a Web-Based Health Literacy, Aligned-Incentive Intervention: Mixed Methods Study
Source: J Med Internet Res. 2019 Oct 17;21(10):e14772. doi: 10.2196/14772 (PMC6823604; doi:10.2196/14772)
Supplement: Multimedia Appendix 3 [file jmir_v21i10e14772_app3.pdf]

If reading medical information is hard for you, please ask a friend or family member to help you.

MedEncentive
Rewarding better health

March 31, 2019

Dear Mary,

This letter is an information therapy prescription from **Dr. Robert Bondurant** (Jane Elizabeth Doe) relating to medical services you received on **March 27, 2019.**

The purpose of this prescription is to help you, in consultation with your doctor, better understand and self-manage your health. Completing this information therapy prescription is easy, and will earn you a reward payment of **$15.00**.

To complete your information therapy prescription, you simply access the MedEncentive website on the Internet. If you have Internet access at home, this is ideal. If not, we recommend contacting your health plan or employer about Internet access at your place of work. You may also consider accessing the Internet at your doctor's office or at the public library.

You will find your information therapy prescription by typing **www.medencentive.com** in the Internet address field. From the MedEncentive homepage, click the "Login" tab, and then select "Patients." To log in, you must enter your username and password. Your username is as follows:

Username: **maryh1**

If you are a first-time user, or have forgotten your password, first enter your username where indicated on the patient login page, and then click the link underneath the password box, and follow the instructions to create or reset your password.

Once logged in, you will find instructions to help you complete your prescription. If you have difficulty reading and understanding medical information, it is recommended that you have someone you trust, like a friend or family member, go online and read this information therapy prescription to you.

Your participation in the MedEncentive Program is voluntary. However, people who participate find the information they read to be very valuable in teaching them about their treatment options and how to better manage their health. Participation in the Program has also been proven to control costs. This will help keep your healthcare coverage affordable.

Please note there is a **two-week time limit** from the date of this letter for you to go online to complete the questionnaires associated with this prescription to be eligible for the financial reward mentioned above.

Once you successfully complete your prescription, expect a check or money transfer from your health plan administrator in a couple of weeks.

- Doctor's name, date of service, and amount of the refund
- Username to secure, HIPAA-compliant website
- Voluntary participation
- Time limit

© 2019 MedEncentive, LLC. All Rights Reserved
