# Supplementary material for: Reduced Hospitalizations, Emergency Room Visits, and Costs Associated with a Web-Based Health Literacy, Aligned-Incentive Intervention: Mixed Methods Study
Source: J Med Internet Res. 2019 Oct 17;21(10):e14772. doi: 10.2196/14772 (PMC6823604; doi:10.2196/14772)
Supplement: Multimedia Appendix 4 [file jmir_v21i10e14772_app4.pdf]

MedEncentive Patient Website

My Activity | Health Articles | Provider Info | My Profile | Logout | Help | Español

Print Article | Save To Favorites | Select Another Article

High Blood Pressure

Topic Overview

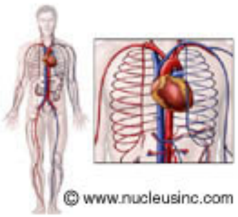

What is high blood pressure?

Blood pressure is a measure of how hard the blood pushes against the walls of your arteries as it moves through your body. It's normal for blood pressure to go up and down throughout the day, but if it stays up, you have high blood pressure. Another name for high blood pressure is hypertension.

Media Gallery
(2 pictures)

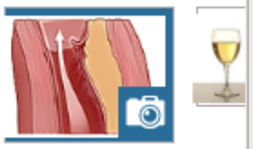

Learn more about How High Blood...

When blood pressure is high, it starts to damage the blood vessels, heart, and kidneys. This can lead to heart attack, stroke, and other problems. High blood pressure is called a "silent killer," because it doesn't usually cause symptoms while it is causing this damage.

Your blood pressure consists of two numbers: systolic and diastolic. Someone with a systolic pressure of 120 and a diastolic pressure of 80 has a blood pressure of 120/80, or "120 over 80." Blood pressure is measured in millimeters of mercury (mm Hg).

- The systolic number shows how hard the blood pushes when the heart is pumping.
- The diastolic number shows how hard the blood pushes between heartbeats, when the heart is relaxed and filling with blood.

An ideal blood pressure for an adult is less than 120/80. High blood pressure is 140/90 or higher. You have high blood pressure if your top number is 140 or higher or your bottom number is 90 or higher, or both. Many people fall into the category called prehypertension, which is in between ideal blood pressure and high blood pressure. People with prehypertension need to make lifestyle changes to bring the blood pressure down and help prevent or delay high blood pressure.

Patient articles supplied by leading educational content developers, like…

healthwise®
for every health decision®

© 2019 MedEncentive, LLC. All Rights Reserved
