# Supplementary material for: Reduced Hospitalizations, Emergency Room Visits, and Costs Associated with a Web-Based Health Literacy, Aligned-Incentive Intervention: Mixed Methods Study
Source: J Med Internet Res. 2019 Oct 17;21(10):e14772. doi: 10.2196/14772 (PMC6823604; doi:10.2196/14772)
Supplement: Multimedia Appendix 5 [file jmir_v21i10e14772_app5.pdf]

MedEncentive Patient Website | My Activity | Health Articles | Provider Info | My Profile | Logout | Help | Español

Questions that pertain to you and this article

Please answer the following questions pertaining to you and this article, and then click the "Continue" button at the bottom of the page.

1. If your doctor prescribed medication for treating your high blood pressure, and you do not have any symptoms, is it okay to stop taking your medicine?

- ○ Yes
- ○ No

Quiz questions are focused on actionable information

2. High blood pressure is the same as:

- ○ Indigestion
- ○ Hypertension
- ○ Headache

3. According to the article, the path that leads to successful lifestyle change includes:

- ☐ Having your own reason for making a change
- ☐ Following the path of least resistance
- ☐ Set long-term goals and short-term goals that you can measure easily
- ☐ Measure improvements to your health
- ☐ Set a path of no return
- ☐ Thinking about what might get in your way, and prepare for slip-ups
- ☐ Getting support from your family, your doctor, and your friends

4. Untreated high blood pressure:

- ○ is not that serious
- ○ can lead to back spasms
- ○ can lead to fatal heart attacks or strokes
- ○ can be resolved quickly
- ○ is not a permanent condition

© 2019 MedEncentive, LLC. All Rights Reserved
