# Supplementary material for: Reduced Hospitalizations, Emergency Room Visits, and Costs Associated with a Web-Based Health Literacy, Aligned-Incentive Intervention: Mixed Methods Study
Source: J Med Internet Res. 2019 Oct 17;21(10):e14772. doi: 10.2196/14772 (PMC6823604; doi:10.2196/14772)
Supplement: Multimedia Appendix 6 [file jmir_v21i10e14772_app6.pdf]

MedEncentive Patient Website | My Activity | Health Articles | Provider Info | My Profile | Logout | Help | Español

Home / **Pending Information Therapy**

Questions that pertain to you and this article

Date Of Service: 11/12/2016
Doctor/Clinician: John Doright Jane Doe
Diagnosis: **i10 / Essential Hypertension**
Article You Read:

To return to the article, click on the title link immediately above

Please share with your doctor how closely you are following the health recommendations contained in this article as you understand them.

- ○ Closely following
- ○ Mostly following
- ◉ Somewhat following
- ○ Mostly not following
- ○ Not following
- ○ Other (please explain):

Responses indicating a level of non-adherence prompt a series of questions to determine why the patient is not willing or able to follow recommendations and what they intend to do

1. Based on your response, please select the reason that best describes your reluctance to being more compliant with the health recommendations:
   - ◉ I do not understand or am confused about the treatment(s)
   - ○ I cannot afford the treatment(s)
   - ○ I am concerned my doctor may have misdiagnosed my condition
   - ○ I believe I am being over-treated for my condition or have been recommended services that are unnecessary. Please explain:
   - ○ I believe I am being under-treated for my condition. Please explain:
   - ○ I do not agree with or am apprehensive about the recommended treatment(s) because:
   - ○ I do not want to follow the health recommendations
2. To become more comfortable with the recommended treatment(s), I plan to:
   - ◉ Return to my doctor for additional consultation
   - ○ Seek advice from another doctor

© 2019 MedEncentive, LLC. All Rights Reserved
