# Supplementary material for: Reduced Hospitalizations, Emergency Room Visits, and Costs Associated with a Web-Based Health Literacy, Aligned-Incentive Intervention: Mixed Methods Study
Source: J Med Internet Res. 2019 Oct 17;21(10):e14772. doi: 10.2196/14772 (PMC6823604; doi:10.2196/14772)
Supplement: Multimedia Appendix 7 [file jmir_v21i10e14772_app7.pdf]

MedEncentive Patient Website

My Activity | Health Articles | Provider Info | My Profile | Logout | Help | Español

Home / Pending Information Therapy / **Other Treatments**

Sharing your responses with your doctor...

In order for you to earn the reward associated with this information therapy prescription, you must agree to make your questionnaire responses available to your physician.

I authorize the release of my questionnaire responses to my doctor.

I agree | **I disagree**

Patients agree to allow their physicians to access their education and adherence responses

Innovator of the Year Winner 2005, 2006, 2008, 2009, 2013, 2014, and 2016 | U.S. Patents 7,925,519 and 9,171,285 | Canadian Patent 2,729,553

© 2019 MedEncentive, LLC. All Rights Reserved. Privacy Statement

© 2019 MedEncentive, LLC. All Rights Reserved
