# Supplementary material for: Reduced Hospitalizations, Emergency Room Visits, and Costs Associated with a Web-Based Health Literacy, Aligned-Incentive Intervention: Mixed Methods Study
Source: J Med Internet Res. 2019 Oct 17;21(10):e14772. doi: 10.2196/14772 (PMC6823604; doi:10.2196/14772)
Supplement: Multimedia Appendix 8 [file jmir_v21i10e14772_app8.pdf]

MedEncentive Patient Website | My Activity | Health Articles ▾ | Provider Info ▾ | My Profile ▾ | Logout | Help | Español ▾

Home / Pending Information Therapy / **Provider Rating**

Article Questionnaire

Date Of Service: 11/12/2016
Doctor/Clinician: John Doright Jane Doe
Diagnosis: i10 / Essential Hypertension
Article(s) You Read: High Blood Pressure

Questions that pertain to your doctor and this article relative to this visit...

1. **Based on the information you read in the article above and your understanding of recommended care for this visit, please indicate your doctor's level of consistency with the recommended care:**

   - (●) **Consistent with recommended care**
   - ( ) **Mostly consistent with recommended care**
   - ( ) **Somewhat consistent with recommended care**
   - ( ) **Mostly inconsistent with recommended care**
   - ( ) **Inconsistent with recommended care**
   - ( ) **Other - Please explain:**

Patients rate how consistent their physicians' care was to the recommended treatments

2. **Based on the information you have read and your understanding of recommended care for this visit, do you believe your doctor has (is):**

   - ( ) **Over-treated(ing) you**
   - (●) **Treated(ing) you just right**
   - ( ) **Under-treated(ing) you**
   - ( ) **I don't know or can't judge how well my doctor is treating me**

[illegible] [illegible]

© 2019 MedEncentive, LLC. All Rights Reserved
