# Supplementary figures and images for: Reduced Hospitalizations, Emergency Room Visits, and Costs Associated with a Web-Based Health Literacy, Aligned-Incentive Intervention: Mixed Methods Study
Source: J Med Internet Res. 2019 Oct 17;21(10):e14772. doi: 10.2196/14772 (PMC6823604; doi:10.2196/14772)

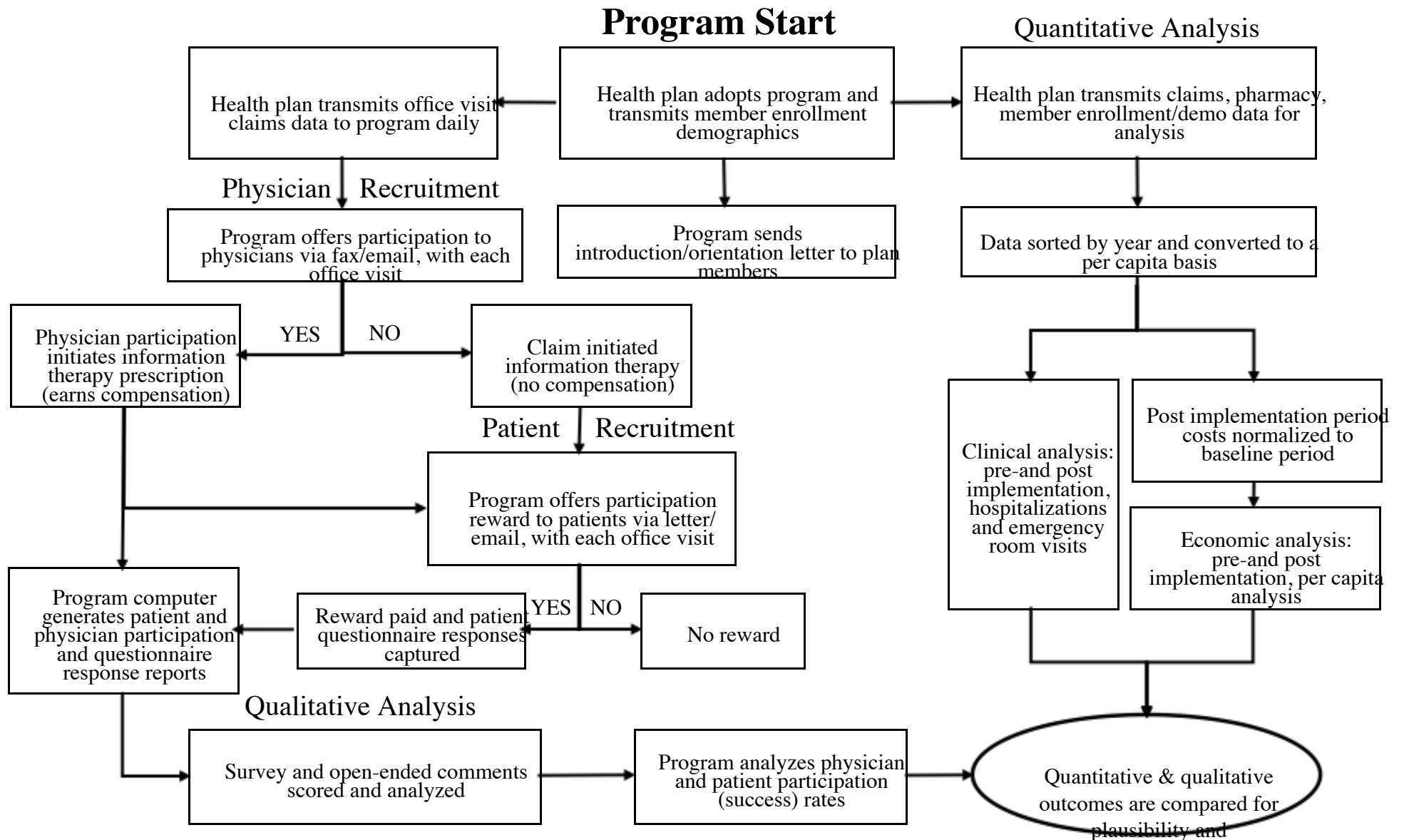

Supplement: Multimedia Appendix 9 [file jmir_v21i10e14772_app9.pdf]

Annual Patient Success Rate by Qtr

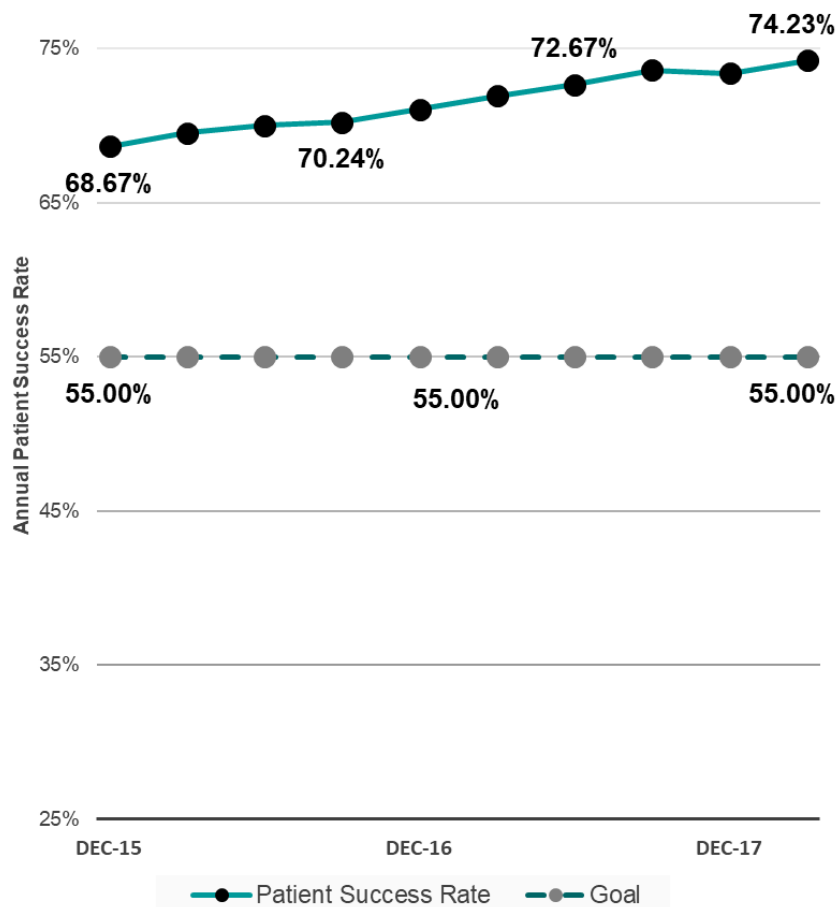

Annual Physician Success Rate by Qtr

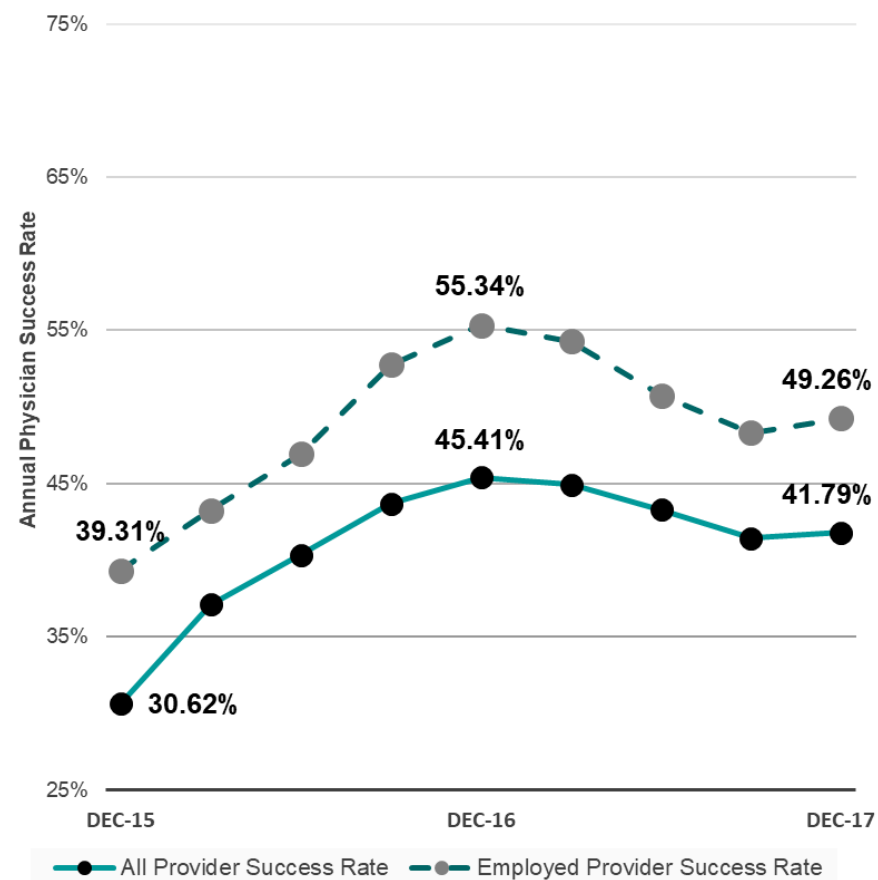

Supplement: Multimedia Appendix 10 [file jmir_v21i10e14772_app10.pdf]
