# Supplementary material for: Reduced Hospitalizations, Emergency Room Visits, and Costs Associated with a Web-Based Health Literacy, Aligned-Incentive Intervention: Mixed Methods Study
Source: J Med Internet Res. 2019 Oct 17;21(10):e14772. doi: 10.2196/14772 (PMC6823604; doi:10.2196/14772)
Supplement: Multimedia Appendix 11 [file jmir_v21i10e14772_app11.pdf]

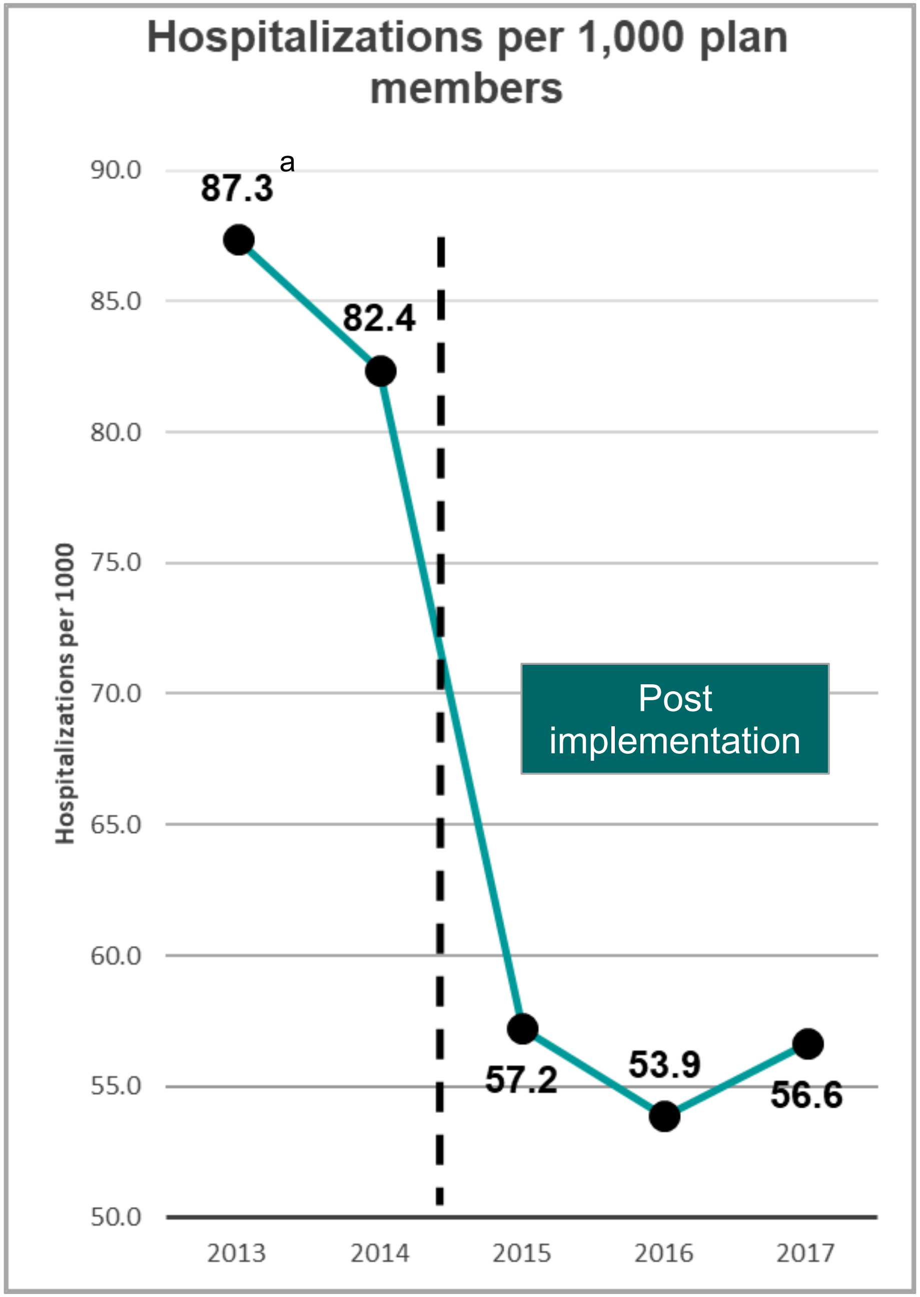

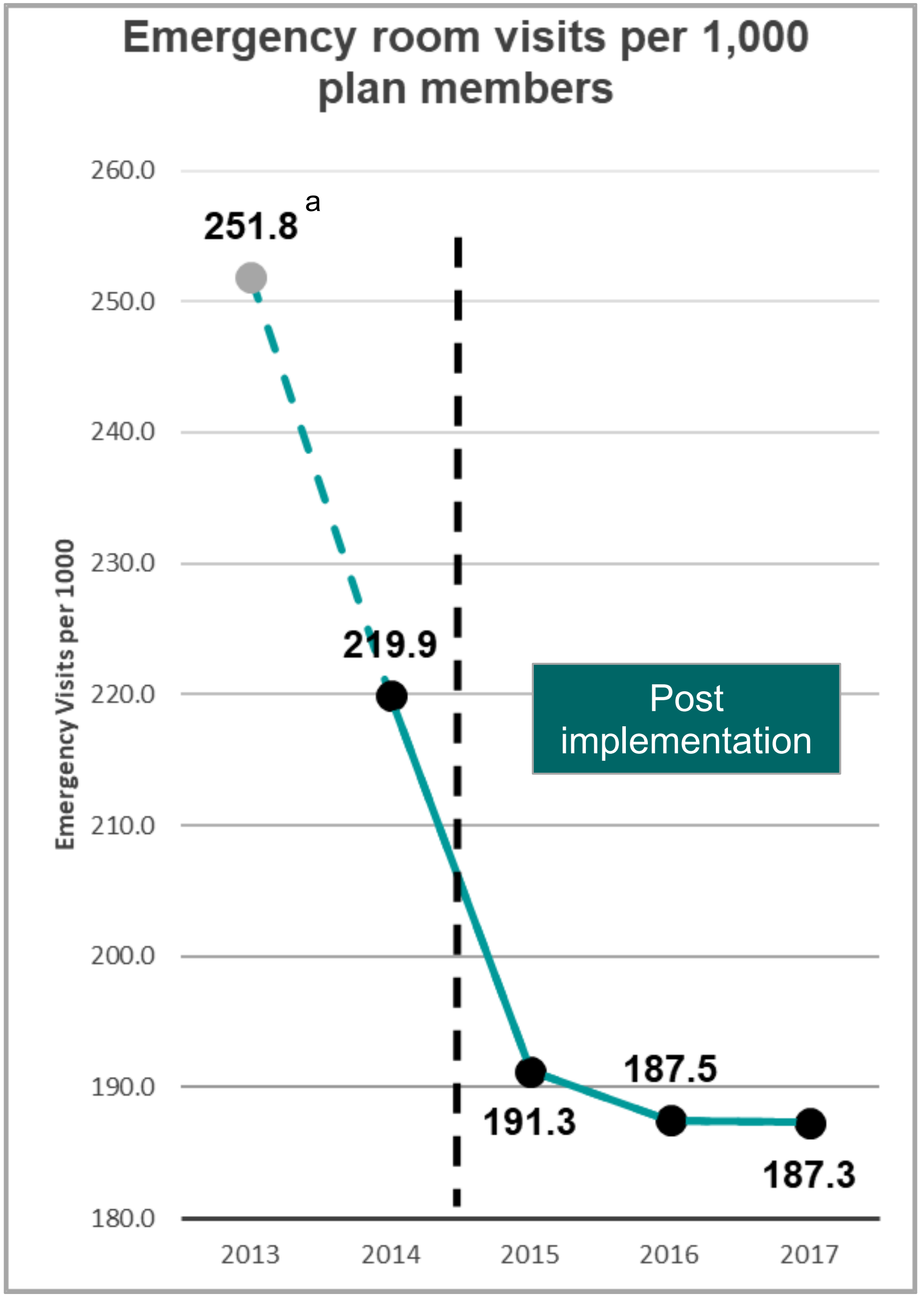

[a] 2012 hospitalizations and emergency room visits were not used as a basis of comparison and are reported for informational purposes.
