# Supplementary material for: Reduced Hospitalizations, Emergency Room Visits, and Costs Associated with a Web-Based Health Literacy, Aligned-Incentive Intervention: Mixed Methods Study
Source: J Med Internet Res. 2019 Oct 17;21(10):e14772. doi: 10.2196/14772 (PMC6823604; doi:10.2196/14772)
Supplement: Multimedia Appendix 12 [file jmir_v21i10e14772_app12.pdf]

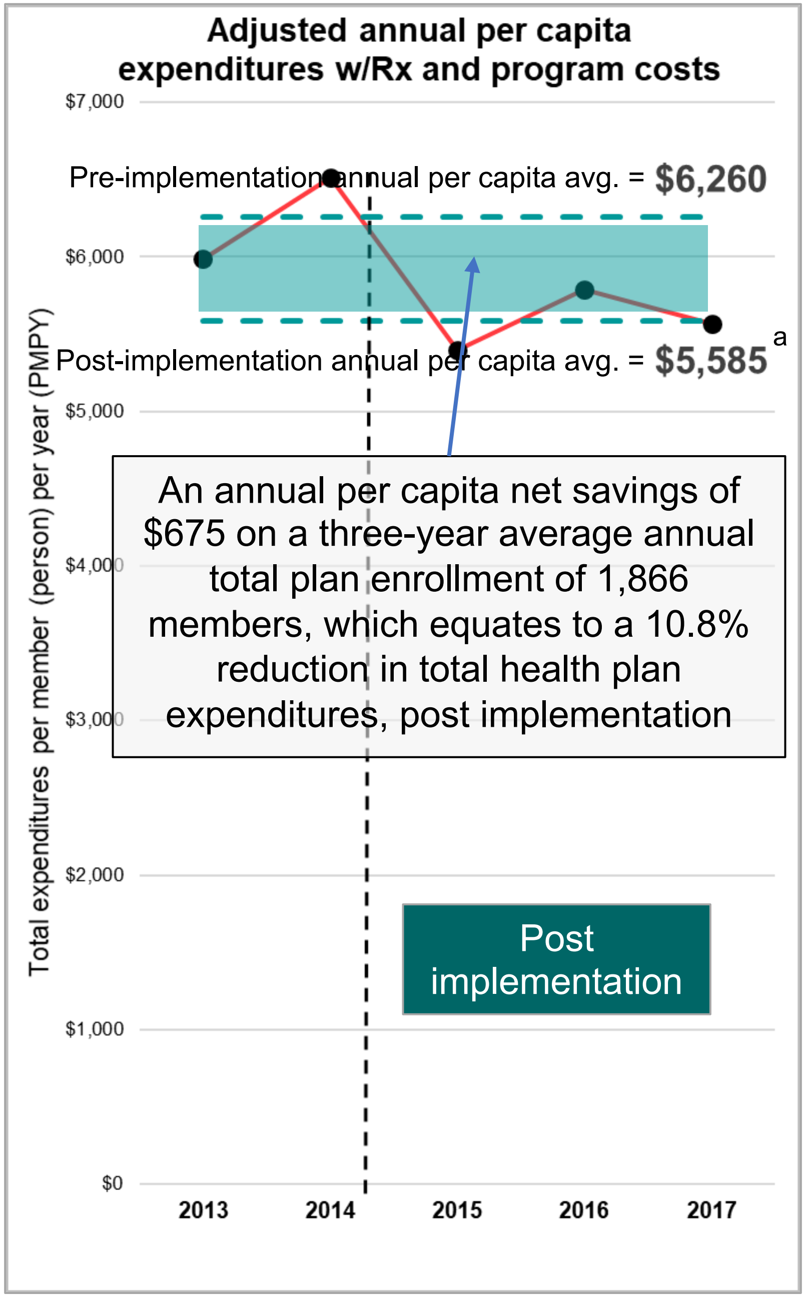

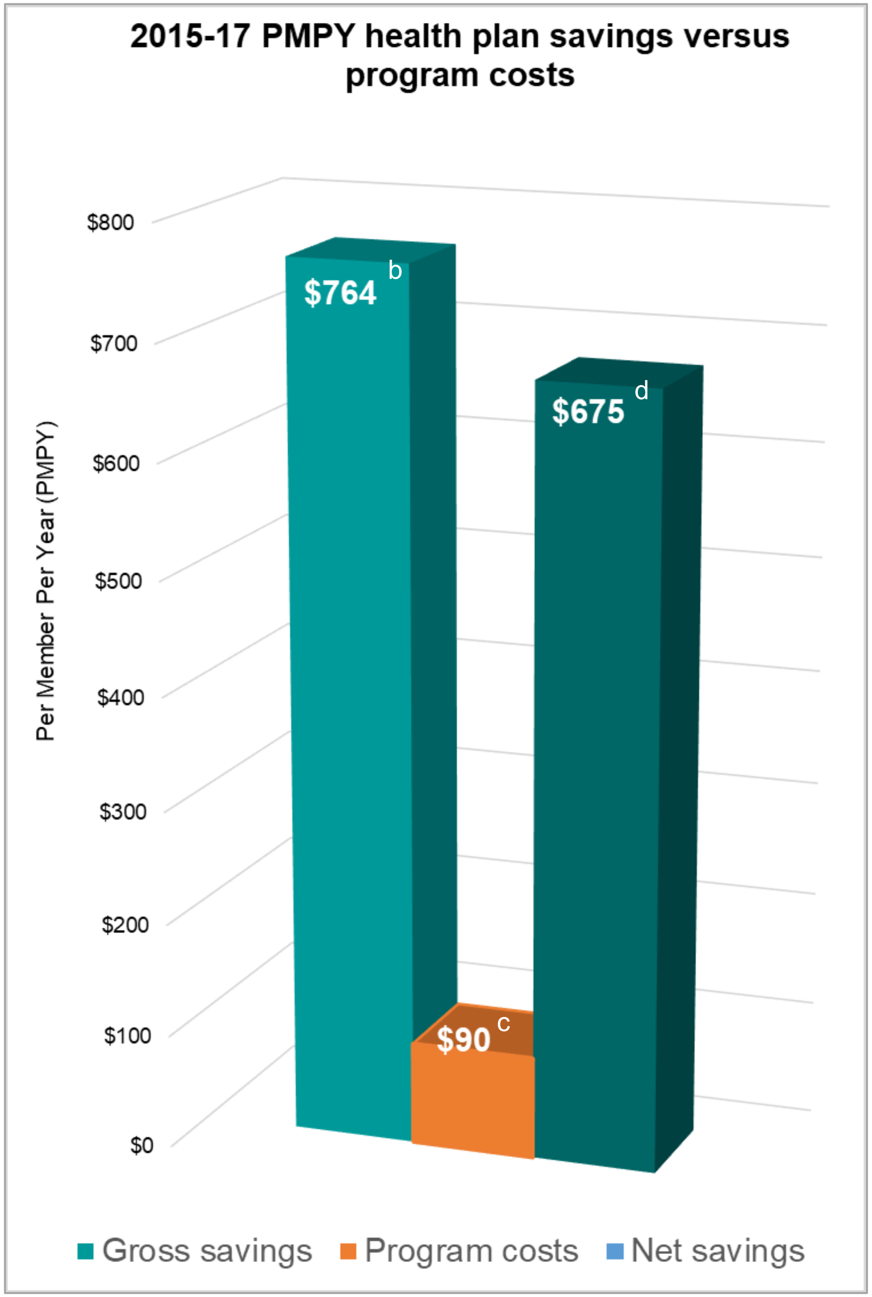

[a] 2015-17 avg. expend.= all medical, Rx and program costs, adjusted to 2013-14 basis

[b] gross savings PMPY = 2015-17 avg. expend. - 2013-14 avg. expend.

[c] total program costs = all patient rewards, physician comp. and program admin fees

[d] net savings PMPY = gross savings – total program costs
